# Sites of Synchronous Distant Metastases, Prognosis, and Nomogram for Small Cell Lung Cancer Patients with Bone Metastasis: A Large Cohort Retrospective Study

**DOI:** 10.1155/2021/9949714

**Published:** 2021-07-28

**Authors:** Zhiyi Fan, Zhangheng Huang, Yuexin Tong, Zhe Zhu, Xiaohui Huang, He Sun

**Affiliations:** ^1^Department of Spine Surgery, Affiliated Hospital of Chengde Medical University, Chengde, Hebei, China; ^2^Department of Hand Surgery, The Second Hospital of Jilin University, Changchun, Jilin, China; ^3^Hangzhou Medical College, Hangzhou, Zhejiang, China

## Abstract

**Background:**

Small cell lung cancer (SCLC) is often associated with metastases at the time of diagnosis, and the bone is one of the most common sites. The primary aim of this study was to investigate the site of synchronous distant metastasis to other organs in SCLC patients with bone metastasis (BM) and develop a robust predictive prognostic model.

**Methods:**

We retrospectively analyzed the data from patients diagnosed with SCLC with BM in the Surveillance, Epidemiology, and End Results database. Univariate and multivariate Cox analyses were used to identify independent prognostic factors. A prognostic nomogram was constructed and evaluated by calibration curves, receiver operating characteristic (ROC) curves, and decision curve analysis (DCA). Then, according to the sites of metastasis and treatment modality, all patients were stratified into several subgroups. The relationship among sites of metastasis, treatment modality, and overall survival was then analyzed.

**Results:**

A total of 6253 patients were included. Independent prognostic factors for SCLC with BM were age, sex, primary site, radiotherapy, chemotherapy, brain metastasis, liver metastasis, and marital status. Calibration, ROC curves, and DCA indicated the excellent performance of the prognostic nomogram. The liver is the most common organ for extraskeletal metastases, followed by the lung. Patients with only BM had the longest mean survival time (9.30 ± 0.31 months). In the subgroup analysis, chemotherapy was an independent prognostic factor for all subgroups. In contrast, radiotherapy showed a positive effect on the prognosis of patients in all subgroups except those with bone and brain metastases and those with bone, lung, and brain metastases.

**Conclusions:**

The prognostic nomogram is expected to be an accurate and personalized tool for predicting the prognosis of SCLC patients with BM. Additionally, the determination of the sites of synchronous extraskeletal metastases and the associated prognosis helps in treatment selection.

## 1. Background

Lung cancer is the leading cause of cancer-related death worldwide, and of all the subtypes, small cell lung cancer (SCLC) accounts for approximately 15% of newly diagnosed lung cancer each year [1, 2]. SCLC is the most aggressive type among the lung cancer subtypes and is often accompanied by metastasis at the time of diagnosis [3, 4]. The incidence of distant metastases at the time of initial diagnosis of SCLC is higher than 60%, and one of the most common sites of metastasis is the bone [5, 6]. Once bone metastasis (BM) occurs, the risk of skeletal-related diseases increases, leading to a decrease in the patient's quality of life and a poor prognosis [7]. The choice of treatment for BM should be based on the patient's expected survival time [8]. Therefore, predicting the survival time of SCLC patients with BM is of great clinical significance.

Many studies have reported the natural history of patients with BM from nonsmall cell lung cancer [9–12]. However, few reports have been published on the prognostic factors and characteristics of SCLC patients with BM. Gong et al. performed a retrospective analysis of 102 SCLC patients with BM at initial diagnosis and suggested that age, number of BM, and occurrence of distant metastases outside the bone were significant prognostic factors [13]. Notably, the limited sample size and the single-center design are obvious weaknesses of that study. Currently, the TNM staging system is widely used to assess the prognosis of cancer patients [14]. However, in addition to TNM staging, it is well known that clinical characteristics such as sex, age, and treatment modality are important factors that may affect the prognosis of cancer patients [15, 16].

To our knowledge, no studies based on large populations to develop a model for predicting the prognosis of SCLC with BM at initial diagnosis have been performed to date. In addition, the sites of synchronous extraskeletal metastases, such as the lungs, brain, and liver, in SCLC patients with BM at initial diagnosis and the associated prognostic outcomes have not been thoroughly investigated. Therefore, the primary aim of this study was to investigate the sites of synchronous distant metastases and the associated prognosis in SCLC patients with BM at initial diagnosis based on data from the Surveillance, Epidemiology, and End Results (SEER) project and to develop an associated predictive model for prognosis. The second objective was to investigate the survival benefits of the treatment modalities (surgery, radiotherapy, and chemotherapy) by stratifying the patients' metastatic sites and treatment modalities.

## 2. Methods

### 2.1. Study Population Selection

**Figure 1:**
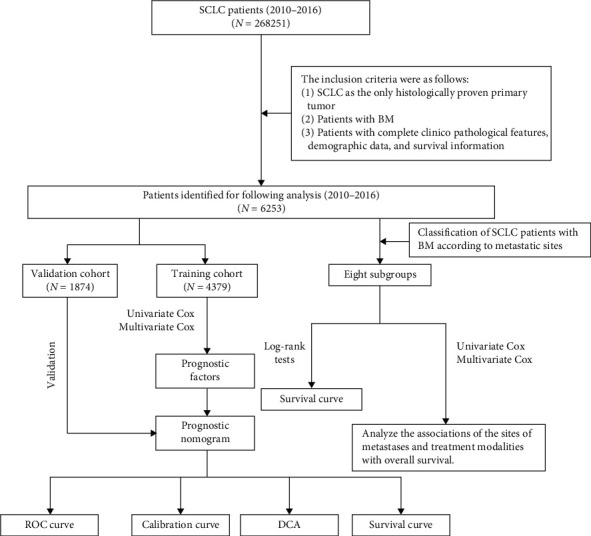
The workflow describing the schematic overview of the project.

The workflow of our study is illustrated in Figure 1. This population-based retrospective study used data from the SEER database. The SEER database consists of 18 population-based cancer registries that collect statistical, oncological, diagnostic, and treatment information for approximately 28% of the population of the United States. This database provides clinical information on cancer patients and greatly facilitates clinical research. Patients diagnosed before 2010 are excluded because the SEER database did not record information on distant metastases (bone, liver, brain, and lung metastases) until 2010. In addition, to ensure adequate follow-up time, patients diagnosed after 2016 are also excluded. Therefore, only SCLC patients diagnosed with BM between 2010 and 2016 were considered in this study.

The inclusion criteria were as follows: (1) SCLC as the only histologically confirmed primary tumor, (2) patients with BM, and (3) patients with complete clinicopathologic features, demographic data, and survival information. Finally, we extracted 6253 SCLC patients with BM at initial diagnosis from 309,056 lung cancer patients. The study population was randomly divided into training and validation cohorts at a 7 : 3 ratio, and the classification process was performed using R software.

### 2.2. Ethics Statement

This study was based on publicly available data from the SEER database (https://seer.cancer.gov/), and a data use agreement was signed. The SEER database does not include personally identifiable information, and because no direct interaction with patients occurred in this study, ethics exemption was obtained from the ethics committee of the local hospital for this study.

### 2.3. Variable Definitions

Based on patient-specific information in the SEER database, we selected 16 variables to identify independent prognostic factors for SCLC with BM, including age, sex, race, primary site, grade, laterality, *T* stage, *N* stage, distant metastatic sites (lung, brain, and liver), surgery, radiotherapy, chemotherapy, insurance status, and marital status. The primary tumor site is defined according to the International Classification of Diseases for Oncology (ICD-O) code: upper lobe of the lung (C34.1), middle lobe of the lung (C34.2), lower lobe of the lung (C34.3), and lung, if not otherwise specified (C34.9). Third edition (ICD-O-3) histology codes, as follows, were used to identify cases with SCLC: 8002 (malignant tumor, small cell type), 8041 (small cell carcinoma, NOS), 8042 (oat cell carcinoma), 8043 (small cell carcinoma, fusiform cell), 8044 (small cell carcinoma, intermediate cell), and 8045 (combined small cell carcinoma). All cases in this study were classified according to the 7^th^ edition of the American Joint Committee on Cancer TNM staging system as grade I (well-differentiated), grade II (moderately differentiated), grade III (poorly differentiated), or grade IV (undifferentiated). Distant organ metastasis is defined by the SEER program as the state of metastasis in distant organs at the time of the first diagnosis of cancer, where the sites of metastasis recorded include the bone, liver, brain, and lung. Regarding marital status, we excluded misleading data on unmarried or domestic partners and then included “unmarried,” “separated,” “single,” and “widowed” all in the unmarried group. Insurance status is divided into insured and uninsured, with both “insured” and “insured/unspecific” included in the insured group. In the survival analysis, the primary endpoint of our study was overall survival (OS), which was defined as the date from diagnosis to death (from any cause) or the date of the last follow-up.

### 2.4. Statistical Analysis

The chi-square test was used for categorical data. The optimal cutoff value of age in terms of OS was determined by *X*-tile software (Yale University, New Haven, CT, USA). To process the data conveniently, we divided the patients into three groups according to age (<66, 67–79, and >79 years) [17]. Univariate and multivariate Cox proportional hazards regression analyses of the training cohort were used to identify independent prognostic factors from which predictive models were constructed. Receiver operating characteristic (ROC) curves and the area under the curve (AUC) were used to evaluate the discrimination of the nomogram. The calibration curve is a graphical display of calibration accuracy and is used to measure the agreement of predicted probabilities with actual survival outcomes. To further assess the benefits and advantages of the predictive model, we used decision curve analysis (DCA). All evaluation processes were conducted 1000 times using bootstrapping. Finally, all patients were divided into high-risk and low-risk groups according to the median risk score, and survival curves were used to verify the prognostic value of the nomogram [18].

Patients with SCLC were classified according to the site of metastasis. A Kaplan–Meier analysis was used to assess survival time for each subgroup of patients, and differences in survival time were determined using the log-rank test. A Cox proportional hazards regression analysis was used to analyze the relationship among metastasis sites, treatment modality, and OS. This study used SPSS 25.0 (NY, USA) and R software (version 3.6.1) for the statistical analysis. In the present study, a *p* value <0.05 (two-sided) indicated statistical significance.

## 3. Results

### 3.1. Baseline Characteristics of the Study Population

Ultimately, 6253 SCLC patients with BM at initial diagnosis were identified from the SEER database and were randomized at a 7 : 3 ratio into a training cohort (*n* = 4379) and a validation cohort (*n* = 1874).  Table 1 summarizes the demographic and clinical characteristics of the SCLC patients with BM. Of all the patients included, 2941 (47.03%) were aged 66–79 years, and the majority of patients were male (55.56%) and white (88.97%). A total of 1408 (22.52%) patients had lung metastases, 1190 (19.03%) had brain metastases, and 3530 (56.45%) had liver metastases. The most common *T* and *N* stages were *T*4 (30.90%) and *N*2 (47.37%). Regarding therapy, 138 (2.21%) of the patients underwent surgery, 4499 (71.95%) received chemotherapy, and 2417 (38.65%) received radiotherapy.

### 3.2. Prognostic Factors for SCLC Patients with BM

Univariate and multivariate Cox proportional hazards regression analyses were performed to screen for prognostic factors. The results of the Cox proportional hazards regression analysis performed for all patients are given in Table 2. In the univariate Cox regression analysis, age, sex, primary site, *T* stage, radiotherapy, chemotherapy, brain metastases, liver metastases, lung metastases, insurance status, and marital status were significantly associated with OS. Finally, the results of the multivariate Cox regression analysis showed that age, sex, primary site, radiotherapy, chemotherapy, brain metastases, liver metastases, and marital status were independent prognostic factors (Table 2). Patients who received radiotherapy and chemotherapy had a lower risk of death with a distribution hazard ratio of 0.801 (95% CI: 0.747–0.860) and 0.272 (95% CI: 0.252–0.294), respectively. In addition, advanced age, male sex, unknown primary site, liver and brain metastases, and unmarried status were associated with a higher risk of death.

### 3.3. Prognostic Nomogram Development and Validation

**Figure 2:**
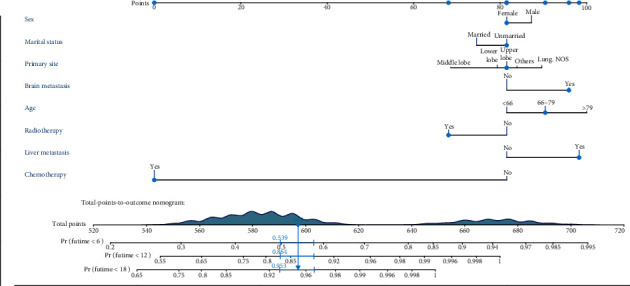
A prognostic nomogram for SCLC patients with BM.

**Figure 3:**
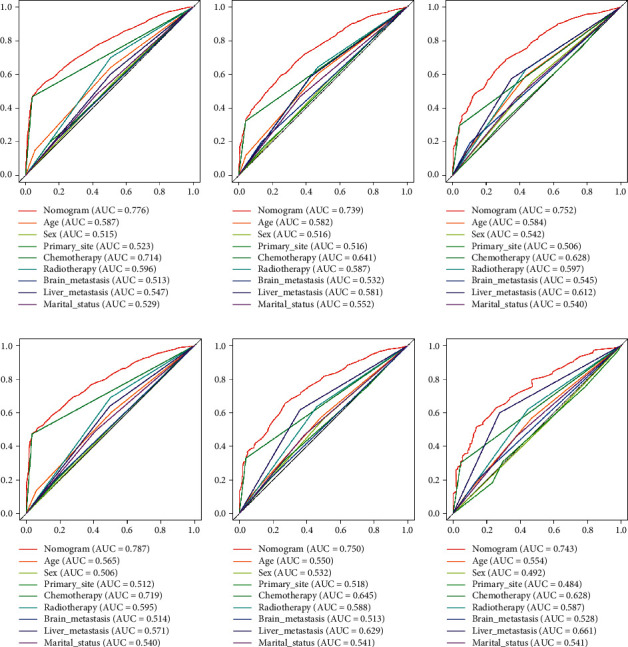
The ROC curves of the nomogram and all independent predictors at 6 (a), 12 (b), and 18 months (c) in the training cohort and at 6 (d), 12 (e), and 18 months (f) in the validation cohort.

**Figure 4:**
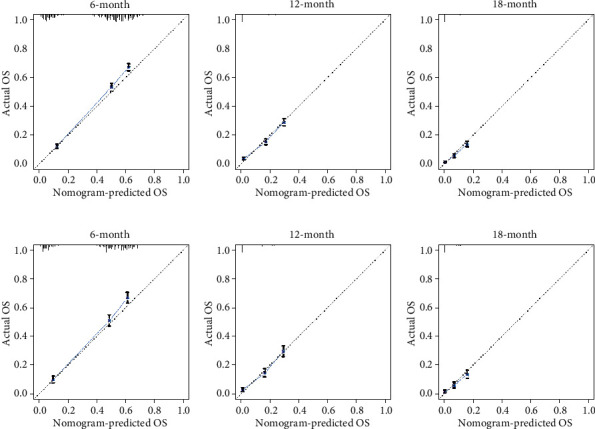
The calibration curves of the nomogram for the prediction of the 6-, 12-, and 18-month OS of patients in the training cohort ((a)–(c)) and validation cohort ((d)–(f)). The *x*-axis represents the nomogram-predicted survival rates, whereas the *y*-axis represents the actual survival rates.

**Figure 5:**
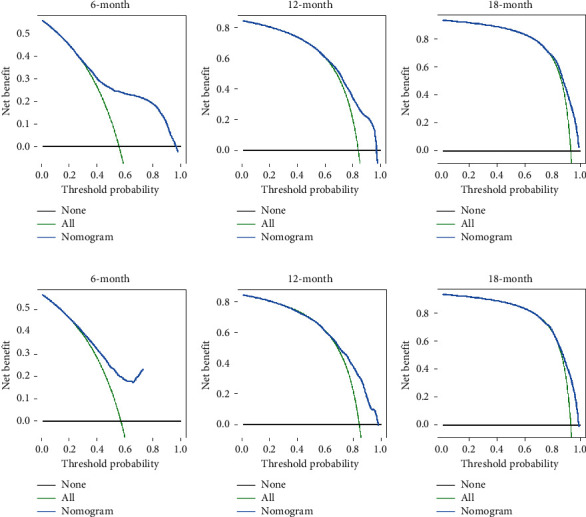
DCA of the nomogram for the survival prediction of SCLC patients with BM. (a) 6-month survival benefit in the training cohort. (b) 12-month survival benefit in the training cohort. (c) 18-month survival benefit in the training cohort. (d) 6-month survival benefit in the validation cohort. (e) 12-month survival benefit in the validation cohort. (f) 18-month survival benefit in the validation cohort. The *x*-axis shows the threshold probability, and the *y*-axis shows the net benefit rate. The black horizontal line indicates that no patients died. The green oblique line indicates the cancer-specific death for all the patients. The red line represents the prognostic nomogram.

Based on the prognostic factors selected in the training cohort, a nomogram was established to predict the OS of SCLC patients with BM (Figure 2). In the prognostic nomogram, values for the individual patient are located along the variable axes, and a line is drawn upward to the points' axis to determine the number of points assigned for each variable. The scores for each variable are then summed to calculate an individual's total risk score, and the 6-, 12-, and 18-month OS are estimated visually by drawing a line from the total score axis to the 6-, 12-, and 18-month survival probability axes. We plotted the ROC curves for the training and validation cohorts and calculated the corresponding AUCs. The AUCs of the nomogram for the 6-, 12-, and 18-month OS reached 0.776, 0.739, and 0.752, respectively, in the training cohort and 0.787, 0.750, and 0.743 in the validation cohort, respectively (Figure 3). In addition, we further compared the difference in the AUC value between the nomogram and all independent prognostic factors, and the results showed that the AUC value of the nomogram was higher than the AUC of all independent factors at 6, 12, and 18 months, both in the training cohort and the validation cohort (Figure 3). As shown in Figure 4, calibration curves were generated to verify the agreement between survival, as predicted by the nomogram, and actual observations. These points are close to a 45-degree diagonal, which indicates that we succeeded in achieving the best agreement between the survival rates predicted by the nomogram and the actual survival rates. DCA showed that the prognostic nomogram has a wider and practical range of threshold probabilities, which significantly increases the net benefit and suggests that this nomogram has high clinical utility in predicting OS in SCLC patients with BM (Figure 5).

### 3.4. Stratification of Risk Groups

**Figure 6:**
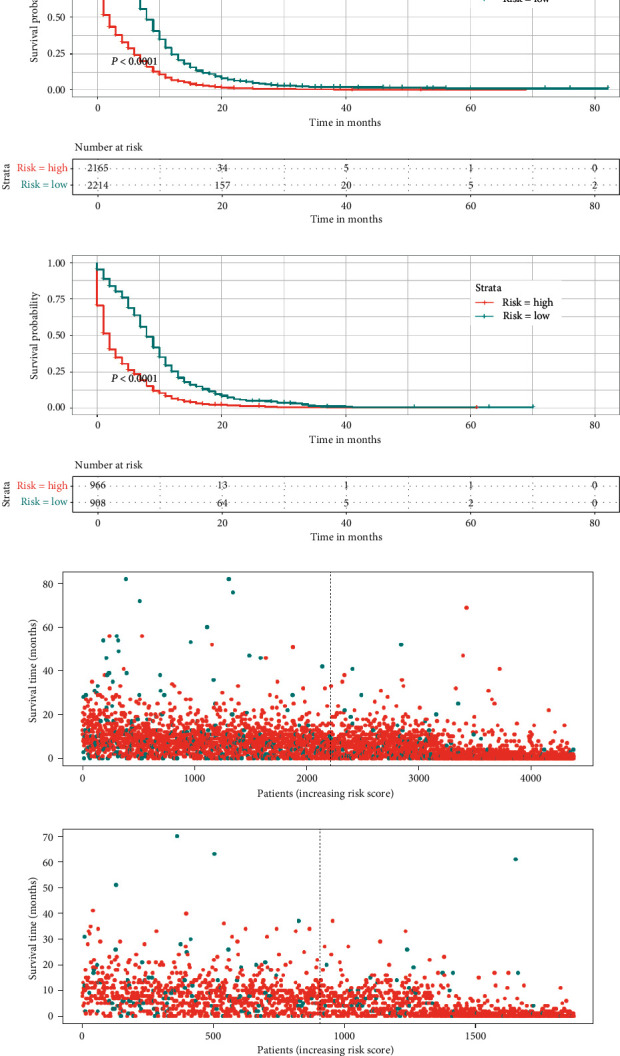
Kaplan–Meier survival analysis of the signature for both the training cohort and the validation cohort. Patients in the training cohort ((a), (c)) and validation cohort ((b), (d)) with a higher risk score demonstrated a worse OS than those with a lower risk score, which suggests the strong predictive ability of the nomogram for the survival outcomes of patients with BM.

Based on the median risk score of patients in the training cohort, all patients, including those in the training and validation cohorts, were divided into low- and high-risk groups. By plotting Kaplan–Meier survival curves, it was easy to observe that patients in the high-risk group exhibited a worse prognosis than those in the low-risk group (Figure 6).

### 3.5. OS Rates (Median, Mean, 1-, 2-, and 5-Year) of SCLC Patients with BM and Different Sites of Metastasis

**Figure 7:**
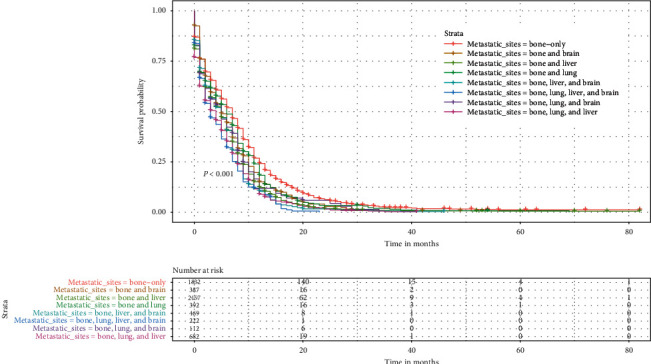
Kaplan–Meier survival curves and log-rank tests showed statistically significant differences in the OS of SCLC patients with BM and different metastatic sites.

Kaplan–Meier survival curve analysis was used to evaluate the prognostic differences among the different metastatic sites. As shown in Table 3 and Figure 7, patients with only BM had the longest mean survival time (9.30 ± 0.31 months), while patients with bone, brain, lung, and liver metastases had the shortest mean survival time (4.86 ± 0.33 months). In patients with two sites of metastasis, those with bone and liver metastases had a worse prognosis than those with bone and brain metastases and bone and lung metastases. Among the patients with metastases at these three sites, those with bone, lung, and liver metastases had the shortest survival time. We can easily see that as the number of metastatic sites outside the bone increases, patients tend to have a shorter survival time. In addition, if a patient has liver metastases, the survival time is shorter regardless of how many other sites of metastasis are present.

### 3.6. Relationship among Sites of Metastasis, Treatment Modality, and OS in SCLC Patients with BM

**Figure 8:**
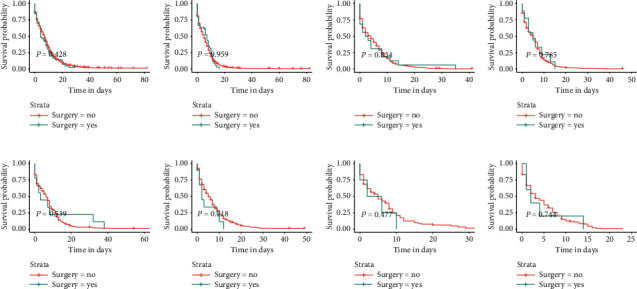
Kaplan–Meier survival curves and log-rank tests for each patient subgroup based on treatment modality. Differences in OS between patients who underwent surgery and those who did not in each subgroup. (a) Bone-only. (b) Bone and liver. (c) Bone, lung, and, liver. (d) Bone, liver, and brain. (e) Bone and lung. (f) Bone and brain. (g) Bone, lung, and brain. (h) Bone, lung, liver, and brain.

**Figure 9:**
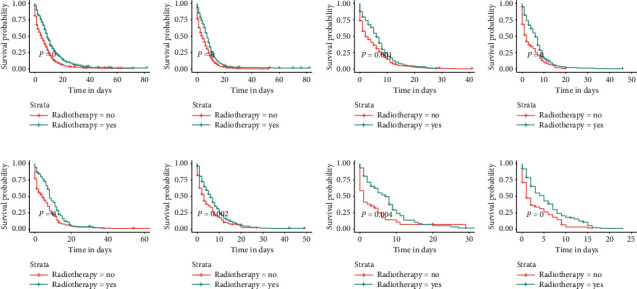
Kaplan–Meier survival curves and log-rank tests for each patient subgroup based on treatment modality. Differences in OS between patients who received radiotherapy and those who did not in each subgroup. (a) Bone-only. (b) Bone and liver. (c) Bone, lung, and, liver. (d) Bone, liver, and brain. (e) Bone and lung. (f) Bone and brain. (g) Bone, lung, and brain. (h) Bone, lung, liver, and brain.

**Figure 10:**
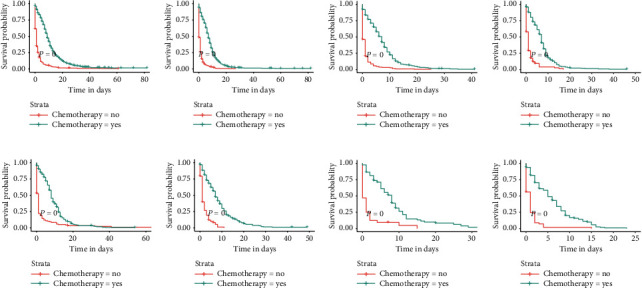
Kaplan–Meier survival curves and log-rank tests for each patient subgroup based on treatment modality. Differences in OS between patients who received chemotherapy and those who did not in each subgroup. (a) Bone-only. (b) Bone and liver. (c) Bone, lung, and, liver. (d) Bone, liver, and brain. (e) Bone and lung. (f) Bone and brain. (g) Bone, lung, and brain. (h) Bone, lung, liver, and brain.

We divided the total cohort into eight subgroups according to the site of metastasis of the patients (bone-only, bone and brain, bone and liver, bone and lung, bone, liver, and brain, bone, lung, and brain, bone, lung, and liver, and bone, lung, liver, and brain). The relationship among the sites of metastasis, treatment modality, and OS is given in Table 4. For all subgroups of patients, chemotherapy was an independent prognostic factor, and all patients who received chemotherapy demonstrated improved OS (all *p* < 0.001). Radiotherapy positively affected OS in some subgroups (all *p* < 0.05), which were the bone-only, bone and liver, bone and lung, bone, liver and brain, and bone, liver, brain, and lung metastases subgroups. Surprisingly, surgery was not an independent prognostic factor in any subgroup of patients. For patients in all subgroups, their OS was not affected regardless of whether they underwent surgery (Figure 8), whereas if they received radiotherapy or chemotherapy, their OS was significantly improved (Figures 9 and 10).

## 4. Discussion

In this study, a nomogram model incorporating age, sex, primary site, radiotherapy, chemotherapy, brain metastasis, liver metastasis, and marital status was constructed to predict the probability of OS of patients with SCLC and BM and was validated using an independent validation cohort. The results show that the nomogram model exhibits good discrimination and accuracy in both the training and validation cohorts. In addition, patients were divided into eight subgroups according to the site of metastasis. We further analyzed the prognosis of each subgroup and investigated the survival benefit of the treatment modality (surgery, radiotherapy, and chemotherapy) on each subgroup using a Cox analysis and K-M survival curve analysis.

Few studies have reported the characteristics and prognosis of SCLC patients with BM. To our knowledge, only two studies have reported the prognostic factors in patients with SCLC with BM at initial diagnosis [13, 19]. However, both the studies by Gong et al. and Kang et al. have significant shortcomings. First, the results are not convincing due to the small sample sizes of 102 and 61 cases, whereas our study included 6253 patients from the SEER database. Since the SEER database is one of the largest open cancer databases globally and covers approximately 28% of the United States population, the results of this study are not the only representative but also highly reliable. Second, some important tumor characteristics and demographic characteristics, such as grade, primary tumor site, laterality, marital status, and insurance status, were not included as variables in the other two studies. Instead, we not only included as many potentially relevant variables as possible but we also constructed a nomogram model based on independent prognostic factors that accurately predicted the prognosis of SCLC patients with BM at initial diagnosis. Compared with the scoring system reported by Gong et al., the nomogram model we constructed has the advantage of visualization of each variable score and individualized survival probability, as well as a more excellent clinical utility [13].

In previous studies, advanced age has been reported to be a poor prognostic factor in patients with SCLC [20–22]. Our study shows that older SCLC patients with BM at initial diagnosis have a higher risk of death. First, this increased risk may be associated with an increased prevalence of degenerative changes and comorbidities in various aspects of organ function [23]. In addition, older patients may be more sensitive to toxicity caused by systemic therapy, whereas younger patients are in good health and can better tolerate the side effects of chemotherapy and radiotherapy [24, 25]. Notably, unmarried status and male sex are poor prognostic factors in SCLC patients with BM. Unmarried patients do not receive psychological and financial support from their spouses, which results in a poorer prognosis [26].

The incidence of distant metastasis at the initial diagnosis of SCLC exceeds 60% [5]. The results of this study show that approximately 70% of SCLC patients initially diagnosed with BM have synchronous distant metastases from other sites. Previous studies that have reported that the liver is the most common organ for extraskeletal metastases, followed by the lung, are consistent with our results [13, 19]. The incidence of liver metastasis was much higher in patients with newly diagnosed SCLC (17.5%) than in patients with nonsmall cell lung cancer (4%) [27]. Our results show that more than 50% of patients have liver metastasis combined with extraskeletal synchronous metastases. We also unexpectedly found that liver metastases were responsible for most of the multisite metastases. This may be explained by the finding that since the liver is an immunosuppressive organ when liver metastases occur, this impedes the liver's immune surveillance of other ongoing metastases [28]. SCLC is a very aggressive malignancy with ubiquitous organ metastases that progress from one organ to another in the majority of patients [29]. Thus, disease progression often leads to an increase in metastatic and a poor prognosis. Liver metastases significantly shorten the survival of lung cancer patients with BM [30]. Our study confirms the findings that liver metastases are always associated with poorer OS. In addition, whenever patients have liver metastases, there is a tendency for a worse prognosis regardless of the number of metastases at other sites, which is in line with previous reports [31]. It is also easy to observe that as the number of metastatic sites outside the bone increases, patients tend to exhibit a shorter survival time (Table 3 and Figure 7) [30, 31]. These results suggest that we need to carefully evaluate the synchronous metastases in extraskeletal organs before treatment is initiated in SCLC patients with BM at initial diagnosis, resulting in more rational and effective treatment decisions.

The main treatment options for SCLC include surgery, chemotherapy, and radiotherapy [32]. The importance of surgery in the treatment of early stage SCLC is widely recognized, but surgery is often not recommended for patients with advanced disease. A growing body of research suggests that surgery is beneficial in prolonging the OS of patients with advanced SCLC [33, 34]. Interestingly, in our study, surgery did not reduce the risk of death according to a multivariate Cox analysis that was performed for all patients and each patient subgroup. For patients with advanced SCLC, either chemotherapy alone or chemotherapy combined with radiotherapy is considered the standard treatment [35]. According to the Cox analysis of all patients, chemotherapy and radiotherapy effectively reduced the risk of death, with HRs of 0.272 and 0.801, respectively. All patients were stratified according to the sites of metastasis and treatment modality to further assess the survival benefit of the treatment modality. In the subgroup analysis, chemotherapy was found to be an independent prognostic factor for each subgroup, whereas radiotherapy showed a positive effect on prognosis for all subgroups except for the bone and brain metastases and bone, lung, and brain metastases subgroups. Through a subgroup analysis performed after the stratification of patients according to treatment modality and metastatic site, we once again confirmed the positive effect of chemotherapy and radiotherapy on prognosis and screened out subgroups of patients whose survival did not improve after radiotherapy. This is important to select a more precise treatment for patients, avoid wasting healthcare resources and guide clinicians in their treatment decisions.

Similar to other studies that have used the SEER database as a data source, this study inevitably has some limitations. First, only specific information on the four metastatic sites was included, and details of metastases were lacking, such as the number of metastatic foci and the sequence in which the organs became metastatic. Second, all metastasis-related information begins with the initial diagnosis, and follow-up information is lacking. Third, the SEER database does not record details of surgical, radiotherapy, and chemotherapy treatments (i.e., surgical procedures, radiation doses, chemotherapy regimens, and chemotherapy sequences). Fourth, the SEER database lacks biomarker information that may be prognostically relevant.

## 5. Conclusions

In summary, this study found that age, sex, primary site, radiotherapy, chemotherapy, brain metastasis, liver metastasis, and marital status influenced the OS of SCLC patients with BM at initial diagnosis. In addition, we constructed a nomogram model for predicting OS. When confronted with an individualized consultation, the nomogram can provide patients with relevant prognostic information and can enhance each patient's prognosis-based decision-making, which is important for the improvement of patient outcomes.

## Figures and Tables

**Table 1:** Demographic and clinical characteristics of SCLC patients with BM.

Variables	Total cohort		Training cohort		Validation cohort	
	*N* = 6253		*N* = 4379		*N* = 1874	
	*n*	%	*n*	%	*n*	%
Age						
<66	2647	42.33	1824	41.65	823	43.92
66–79	2941	47.03	2082	47.55	859	45.84
>79	665	10.63	473	10.80	192	10.25

Race						
Black	419	6.70	292	6.67	127	6.78
Others	267	4.27	191	4.36	76	4.06
White	5563	88.97	3894	88.92	1669	89.06
Unknown	4	0.06	2	0.05	2	0.11

Sex						
Female	2779	44.44	1948	44.49	831	44.34
Male	3474	55.56	2431	55.51	1043	55.66

Primary site						
Upper lobe	2857	45.69	2018	46.08	839	44.77
Middle lobe	219	3.50	158	3.61	61	3.26
Lower lobe	1260	20.15	865	19.75	395	21.08
Lung, NOS	1120	17.91	770	17.58	350	18.68
Others	797	12.75	568	12.97	229	12.22

Grade						
I	7	0.11	6	0.14	1	0.05
II	8	0.13	6	0.14	2	0.11
III	457	7.31	317	7.24	140	7.47
IV	757	12.11	526	12.01	231	12.33
Unknown	5024	80.35	3524	80.48	1500	80.04

Laterality						
Bilateral	89	1.42	58	1.32	31	1.65
Left	2519	40.28	1771	40.44	748	39.91
Right	3286	52.55	2297	52.45	989	52.77
Unknown	359	5.74	253	5.78	106	5.66

*T* stage						
*T*1	434	6.94	292	6.67	142	7.58
*T*2	1145	18.31	801	18.29	344	18.36
*T*3	1094	17.50	744	16.99	350	18.68
*T*4	1932	30.90	1390	31.74	542	28.92
Unknown	1648	26.36	1152	26.31	496	26.47

*N* stage						
*N*0	499	7.98	344	7.86	155	8.27
*N*1	306	4.89	227	5.18	79	4.22
*N*2	2962	47.37	2100	47.96	862	46.00
*N*3	1455	23.27	992	22.65	463	24.71
Unknown	1031	16.49	716	16.35	315	16.81

Radiotherapy						
No	3836	61.35	2696	61.57	1140	60.83
Yes	2417	38.65	1683	38.43	734	39.17

Chemotherapy						
No	1754	28.05	1220	27.86	534	28.50
Yes	4499	71.95	3159	72.14	1340	71.50

Surgery						
No	6115	97.79	4279	97.72	1836	97.97
Yes	138	2.21	100	2.28	38	2.03

Brain metastasis						
No	5063	80.97	3550	81.07	1513	80.74
Yes	1190	19.03	829	18.93	361	19.26

Liver metastasis						
No	2723	43.55	1930	44.07	793	42.32
Yes	3530	56.45	2449	55.93	1081	57.68

Lung metastasis						
No	4845	77.48	3380	77.19	1465	78.18
Yes	1408	22.52	999	22.81	409	21.83

Insurance status						
Uninsured	203	3.24	140	3.20	63	3.36
Insured	6050	96.75	4239	96.80	1811	96.64

Marital status						
Unmarried	2833	45.31	1982	45.26	851	45.41
Married	3420	54.69	2397	54.74	1023	54.59

SCLC, small cell lung cancer; BM, bone metastasis.

**Table 2:** Univariate and multivariate Cox analyses in SCLC patients with BM.

		Univariate Cox analysis				Multivariate Cox analysis			
		HR	95% CI		*P*	HR	95% CI		*P*
Age									
	<66								
	66–79	1.216	1.138	1.300	≤0.01	1.155	1.080	1.235	≤0.01
	>79	1.865	1.678	2.073	≤0.01	1.344	1.204	1.500	≤0.01

Race									
	Black								
	Others	1.111	0.917	1.346	0.281				
	White	1.058	0.935	1.198	0.372				
	Unknown	0.670	0.167	2.692	0.572				

Sex									
	Female								
	Male	1.083	1.018	1.153	0.012	1.099	1.031	1.172	≤0.01

Primary site									
	Upper lobe								
	Middle lobe	0.804	0.675	0.959	0.015	0.806	0.676	0.961	0.016
	Lower lobe	1.021	0.939	1.111	0.621	0.971	0.892	1.056	0.487
	Lung, NOS	1.159	1.063	1.264	≤0.01	1.130	1.036	1.232	≤0.01
	Others	0.988	0.896	1.090	0.811	1.025	0.929	1.131	0.618

Grade									
	I								
	II	2.220	0.677	7.275	0.188				
	III	1.926	0.858	4.325	0.112				
	IV	1.883	0.842	4.214	0.123				
	Unknown	1.994	0.895	4.444	0.091				

Laterality									
	Bilateral								
	Left	0.923	0.702	1.213	0.565				
	Right	0.927	0.706	1.218	0.588				
	Unknown	1.128	0.837	1.520	0.430				

*T* stage									
	*T*1								
	*T*2	1.075	0.938	1.233	0.299				
	*T*3	1.156	1.007	1.327	0.040				
	*T*4	1.148	1.009	1.306	0.036				
	Unknown	1.184	1.033	1.356	0.015				

*N* stage									
	*N*0								
	*N*1	0.983	0.829	1.166	0.847				
	*N*2	1.013	0.902	1.139	0.825				
	*N*3	1.015	0.895	1.151	0.814				
	Unknown	1.053	0.910	1.220	0.487				

Surgery									
	No								
	Yes	1.018	0.830	1.248	0.864				

Radiotherapy									
	No								
	Yes	0.676	0.633	0.721	≤0.01	0.801	0.747	0.860	≤0.01

Chemotherapy									
	No								
	Yes	0.249	0.231	0.268	≤0.01	0.272	0.252	0.294	≤0.01

Brain metastasis									
	No								
	Yes	1.162	1.073	1.259	≤0.01	1.271	1.168	1.384	≤0.01

Liver metastasis									
	No								
	Yes	1.304	1.224	1.389	≤0.01	1.309	1.227	1.395	≤0.01

Lung metastasis									
	No								
	Yes	1.154	1.072	1.243	≤0.01				

Insurance status									
	Uninsured								
	Insured	0.755	0.634	0.900	≤0.01				

Marital status									
	Unmarried								
	Married	0.855	0.804	0.911	≤0.01	0.895	0.839	0.954	≤0.01

SCLC, small cell lung cancer; BM, bone metastasis.

**Table 3:** OS rates (median, mean) of SCLC patients with BM in different metastatic sites.

Metastatic sites	Median survival (months)	Mean survival (months)
Bone-only	7.00 ± 0.25	9.30 ± 0.31
Bone and brain	5.00 ± 0.52	7.12 ± 0.40
Bone and liver	5.00 ± 0.20	6.45 ± 0.20
Bone and lung	6.00 ± 0.53	7.63 ± 0.49
Bone, liver, and brain	5.00 ± 0.45	5.77 ± 0.29
Bone, lung, and brain	5.00 ± 1.04	6.69 ± 0.74
Bone, lung, and liver	4.00 ± 0.39	5.34 ± 0.24
Bone, lung, liver, and brain	3.00 ± 0.44	4.86 ± 0.33

OS, overall survival; SCLC, small cell lung cancer; BM, bone metastasis.

**Table 4:** Univariate and multivariate Cox regression for overall survival based on treatment modalities and metastatic sites.

Groups (*n*)	Variables	Level	*n*	Univariate	*P* value	Multivariate	*P* value
				HR (95% CI)		HR (95% CI)	
Bone-only (1832)	Surgery	No	1786				
		Yes	46	1.123 (0.829–1.520)	0.454		
	Radiotherapy	No	1117				
		Yes	715	0.613 (0.554–0.679)	≤0.01	0.784 (0.705–0.873)	≤0.01
	Chemotherapy	No	485				
		Yes	1347	0.239 (0.212–0.268)	≤0.01	0.262 (0.231–0.297)	≤0.01

Bone and brain (387)	Surgery	No	377				
		Yes	10	1.471 (0.758–2.856)	0.254		
	Radiotherapy	No	94				
		Yes	293	0.698 (0.546–0.893)	≤0.01		
	Chemotherapy	No	97				
		Yes	290	0.233 (0.178–0.304)	≤0.01	0.233 (0.178–0.304)	≤0.01

Bone and liver (2157)	Surgery	No	2118				
		Yes	39	0.992 (0.713–1.380)	0.962		
	Radiotherapy	No	1553				
		Yes	604	0.675 (0.611–0.745)	≤0.01	0.794 (0.717–0.878)	≤0.01
	Chemotherapy	No	608				
		Yes	1549	0.229 (0.205–0.254)	≤0.01	0.243 (0.218–0.271)	≤0.01

Bone and lung (392)	Surgery	No	383				
		Yes	9	0.822 (0.420–1.610)	0.567		
	Radiotherapy	No	265				
		Yes	127	0.654 (0.523–0.817)	≤0.01	0.777 (0.618–0.976)	0.030
	Chemotherapy	No	119				
		Yes	273	0.345 (0.273–0.436)	≤0.01	0.367 (0.289–0.467)	≤0.01

Bone, liver, and brain (469)	Surgery	No	460				
		Yes	9	0.919 (0.475–1.781)	0.803		
	Radiotherapy	No	165				
		Yes	304	0.616 (0.504–0.752)	≤0.01	0.785 (0.637–0.968)	0.024
	Chemotherapy	No	130				
		Yes	339	0.247 (0.195–0.311)	≤0.01	0.264 (0.208–0.336)	≤0.01

Bone, lung, and brain (112)	Surgery	No	108				
		Yes	4	1.402 (0.513–3.830)	0.510		
	Radiotherapy	No	34				
		Yes	78	0.557 (0.361–0.859)	≤0.01		
	Chemotherapy	No	32				
		Yes	80	0.265 (0.168–0.419)	≤0.01	0.210 (0.127–0.348)	≤0.01

Bone, lung, and liver (682)	Surgery	No	666				
		Yes	16	0.952 (0.576–1.573)	0.848		
	Radiotherapy	No	522				
		Yes	160	0.751 (0.626–0.903)	≤0.01		
	Chemotherapy	No	224				
		Yes	458	0.259 (0.216–0.311)	≤0.01	0.262 (0.218–0.314)	≤0.01

Bone, lung, liver, and brain (222)	Surgery	No	217				
		Yes	5	1.145 (0.470–2.786)	0.766		
	Radiotherapy	No	86				
		Yes	136	0.594 (0.447–0.789)	≤0.01	0.681 (0.508–0.914)	0.011
	Chemotherapy	No	59				
		Yes	163	0.246 (0.175–0.345)	≤0.01	0.261 (0.183–0.373)	≤0.01

## Data Availability

The dataset from the SEER database generated and/or analyzed during the current study is available in the SEER dataset repository (https://seer.cancer.gov/).

## Abbreviations

SCLC: Small cell lung cancer
BM: Bone metastasis
SEER: Surveillance, Epidemiology, and End results
OS: Overall survival
ROC: Receiver operating characteristic
AUC: Area under the curve
DCA: Decision curve analysis.
